# Prevalence of Bovine Mastitis Pathogens in Bulk Tank Milk in China

**DOI:** 10.1371/journal.pone.0155621

**Published:** 2016-05-17

**Authors:** Yanliang Bi, Ya Jing Wang, Yun Qin, Roger Guix Vallverdú, Jaime Maldonado García, Wei Sun, Shengli Li, Zhijun Cao

**Affiliations:** 1 State Key Laboratory of Animal Nutrition, Beijing Engineering Technology Research Center of Raw Milk Quality and Safty Control, College of Animal Science and Technology, China Agricultural University, Beijing, China; 2 Laboratorios Hipra S.A., Amer Giron, Spain; The University of Melbourne, AUSTRALIA

## Abstract

The objectives of this study were to estimate the herd prevalence of major mastitis pathogens in bulk tank milk (BTM) in China dairy herds, to determine the relationship between the presence of mastitis pathogens and bulk tank milk somatic cell counts (BTSCC), and to investigate the impact of different dairy cattle farming modes and region on bacterial species. BTM samples collected from 894 dairy herds in China were examined for the presence of mastitis pathogens. The Flinders Technology Associates (FTA) cards were used for BTM sample collection, storage, and transportation and bacterial DNA amplification by real-time PCR. Among contagious pathogens, *Staphylococcus aureus*, *Streptococcus agalactiae*, and *Streptococcus dysgalactiae* were detected in 50.1, 92.2, and 72.3% of the 894 BTM samples, respectively. Among environmental pathogens, *E*. *coli*, *Streptococcus uberis*, *Enterococcus* spp., *Klebsiella* spp., *Serratia marcescens*, *Corynebacterium bovis*, and *Arcanobacterium pyogenes* were detected in 28.6, 8.9, 35.7, 20.0, 1.3, 17.0, and 67.2% of the BTM samples, respectively. Staphylococcal β-lactamase gene was detected in 61.7% of the BTM samples. The presence of *Staphylococcus aureus* and *Arcanobacterium pyogenes* were significantly associated with high BTSCC, respectively. Significant differences were found in presence of *Staphylococcus aureus*, *Streptococcus agalactiae*, and *Streptococcus dysgalactiae* in BTM sampled from the small household farms, dairy-farming communities, and large-scaled dairy farms. There were significant differences in the presence of *Streptococcus agalactiae*, *Streptococcus dysgalactiae*, *Arcanobacterium pyogenes*, staphylococcal β-lactamase gene, *Staphylococcus* spp., *Klebsiella* spp., *Enterococcus* spp., and *Streptococcus uberis* in BTM among Inner Mongolia, Heilongjiang, and Hebei province. In conclusion, contagious mammary pathogens are predominated among pathogens in BTM samples in China.

## Introduction

Bovine mastitis is the inflammation of the mammary gland which can be caused by several bacterial species or species groups. Previous studies showed that the overwhelming majority of clinical and subclinical mastitis were caused by approximately 10 bacterial species or species groups [1–3]. Mastitis pathogens are broadly classified as contagious and environmental pathogens. *Staphylococcus aureus*, *Streptococcus agalactiae*, and *Streptococcus dysgalactiae* are considered as contagious pathogens, which adapt to the environment of mammary gland and can potentially be spread from cow to cow during milking. *Streptococcus uberis*, *Enterococcus* spp., coliforms, *Arcanobacterium pyogenes*, and coagulase-negative staphylococci (CNS) are considered as environmental pathogens, which are described as opportunistic pathogens of the mammary gland and can potentially be transferred from the contaminated environment to the mammary gland of a cow during milking [1, 4].

Early identification of the prevalence and distribution of causative pathogens is of the most importance to effectively prevent diseases and to guide treatment [5–7]. Bulk tank milk (BTM) analysis has been used as a useful monitoring tool to assess milk quality and mastitis pathogens for many years [8–10]. The BTM sample test is less expensive, faster, and more convenient compared with testing quarter milk samples [11–12]. However, in contrast to quarter milk sample test, results of BTM sample test not only reveal the presence of mammary pathogens in a herd but also reflect milk contamination from the teat surface and the environment [1, 7].

The real-time PCR, which has predefined cycle threshold (Ct) values and internal controls allowing differentiation between truly positive and false negative results, has proved useful to identify mastitis pathogens [13–14]. It is based on identification of bacterial genome sequences, and can detect growth-inhibited and dead bacteria within 3 to 4 hours [14]. The real-time PCR assay has high sensitivity and specificity for diagnosis of mastitis pathogens in BTM. Mweu et al. [15] reported that, at a cycle threshold cut-off value of 40, the sensitivity and specificity of the real-time PCR used for screenings of BTM samples for *S*. *agalactiae* were 95.2 and 98.8%, respectively.

Flinders Technology Associates (FTA) cards technique is a well-established method for collection and storage of bacterial DNA samples [16–17]. FTA cards comprise cotton-based cellulose paper, impregnated with proprietary chemicals that serve to lyse cells, denature proteins, stabilize and preserve nucleic acids within the matrix fibre. By doing so the nucleic acids in the samples are immobilized thus allowing to transport easily and long-term storage at room temperature [18]. FTA cards have been used for preparation of DNA or RNA from a variety of biological materials, including blood, saliva, animal and plant tissues, and various pathogens coming from human, animal and plant samples [19–23]. In a previous report [24], raw and mastitis milk, as well as dairy process and product samples were tested using both FTA cards and PCR to detect *S*. *aureus* among other bacteria.

A considerable number of studies of mastitis pathogens in BTM have been published internationally [1, 3, 10]. To our knowledge, there are no scientific reports on the prevalence of major mastitis pathogens in BTM in China. The objectives of this study were 1) to use real time PCR to estimate the prevalence of mastitis pathogens in BTM from Chinese dairy herds, 2) to determine the relationships between mastitis pathogens and bulk tank milk somatic cell counts (BTSCC), and 3) to investigate the effect of geographic region and different dairy cattle farming modes on bacterial species prevalence in BTM.

## Materials and Methods

### Participants

All experimental protocols were reviewed and approved by the Animal Care Committee of China Agricultural University. Experimental animals care and use for this experiments were performed following the established guidelines of the College of Animal Science and Technology, China Agricultural University.

The study was conducted in the top 3 milk-producing provinces (Hebei, Heilongjian and Inner Mongolia) of China. In 2012, 52.3% of the total number of dairy cattle and 45.6% of the total milk output in China came from these 3 provinces (National Bureau of Statistics of China 2013). The 3 provinces are all located in the northern region of China and also have the longest history of dairy farming among all the Chinese provinces. Hebei province (E 113°27'~119°50', N 36°05'~42°40') is located in the eastern plain of China. Heilongjiang province (E 121°11'~135°05', N 43°25'~ 53°33') belongs to the plain of northern cold region. Inner Mongolia (E 97°10'~126°02', N 37°30~53°20') is located in the plateau region of northwest China. Herd selection was supported by provincial bureau of animal husbandry of the three provinces who provided the dairy herds information. 1,000 herds were randomly selected from the three provinces (400 herds from Mongolia, 300 herds from Heilongjiang province, 300 herds from Hebei province) and were invited to participate in the study. Dairy producers who participated in the study were solicited by provincial bureau of animal husbandry through their announcements about the study. Dairy producers who provided written response to the invitation were included in the study. A total of 894 dairy herds from Inner Mongolia (375 herds), Heilongjiang (243 herds) and Hebei (275 herds) responded in writing to the invitation and participated in this study (with approximately 228,000 lactating cows). The proportion of lactating cows in this study are 6.5% in Inner Mongolia, 5.9% in Heilongjiang, and 8.4% in Hebei, respectively. The average lactating cow herd size was 255 cows and ranged from 5 to 6563 cows. In China, BTSCC and bacteria counts are routinely analyzed by milk processing factories which collect milk from dairy farms/communities. Out of the 894 dairy herds, 311 herds (161 herds from Mongolia, 91 herds from Heilongjiang province, 59 herds from Hebei province) were not willing to share their data of BTSCC. Therefore, a total of 583 dairy herds’ data of BTSCC were collected.

The dairy cattle farming modes were classified into three types according to their scale and organization in China, including small household farms, dairy-farming communities (cooperative dairy farms) and large-scaled dairy farms. Small household farm refers to the family farm which normally has less than 30 dairy cattle, which are not grouped, kept in the backyard and milked at the milk collection station close by. This dairy cattle farming mode is relatively simple and the milk yield on the farm is low. The dairy-farming community is a transitional form between the small household farm and the large-scaled dairy farm. In general, the number of dairy cattle is greater than 100 in a dairy-farming community. The dairy cattle owned by different farmers are raised in different barns on one community farm and separated according to ownership rather than grouped and raised according to the age and the physiological cycle. In a dairy-farming community, different dairy cattle belonged to different farmers are milked in one milking parlour and unified management methods can not be achieved. A large-scaled dairy farm refers to a dairy farm owning over 200 dairy cattle, where the cattle are grouped, bred and managed according to their ages and physiological cycles. Unified management methods are applied in this dairy cattle farming mode. Relatively, the large-scaled dairy farms are better mechanized, and raw milk quality and average milk yield are higher than that of other two dairy cattle farming modes. In this study, each milk collection station served as one dairy herd and provides one BTM sample, so did each dairy-farming community. Among the 894 dairy herds, the proportion of BTM samples came from small household farms, dairy-farming communities and large-scaled dairy farms were 47.9, 25.3, and 26.8%, respectively.

### BTM sample and data collections

In the period of April 2012 to October 2013, a total of 894 BTM samples from Mongolia, Heilongjiang and Hebei provinces were collected. Sampling was conducted by 3 trained university students who followed a standardized protocol, and used a standard collection kit provided in advanced. Briefly, the milk in the bulk tank was mixed thoroughly before collection, and a sample was taken from the top of the bulk tank using gloves and a sterile disposable plastic pipet. Approximately 100 μl of each sample were spotted onto the matrix area of a FTA card aseptically and were allowed to air-dry for 1 h at room temperature. After air-dried, FTA cards were placed in hermetic polythene bags containing a desiccant, and were submitted to the molecular biology laboratory (Laboratorios Hipra S.A., Beijing, China).

The information of sampling sites, dairy cattle farming modes and BTSCC of herds were recorded. In China, BTSCC is routinely analyzed by milk processing factories. In this study, BTSCC was determined with a Fossomatic cell counter instrument (Foss Electric A/S, Hillerød, Denmark).

### Milk sample preparation and DNA extraction

For DNA elution, the entire inoculation area of each FTA card (round shape) was removed and cut into small fragments using sterile scissors and forceps. To avoid cross-contamination, the scissors and forceps used to handle the inoculation were changed between samples and were disinfected with high temperature alcohol burner. The small fragments were transferred in a 2 ml sterile polypropylene tube with safety latch. 1 ml of PCR water was transferred to each tube containing samples, caped and vortexed for 15 seconds, and then incubated for 20 minutes in a dry block at 100°C. After incubation, the tube was vortexed for 15 seconds, centrifuged at low speed, and thus the supernatant was obtained.

Liquid samples (supernatants) were processed as if they had been fresh milk samples. DNA in each sample was extracted and purified using the spin column-based nucleic acid purification reagents and the manufacturer’s instructions included in the assay kit (PathoProof™ Mastitis Complete-12 KingFisher™ Kit, Thermo Fisher Scientific, Beijing, China). The extracted DNA was stored at −20°C until further analysis.

### Real-Time PCR analysis

The Real-Time PCR tests were performed using the reagents and protocol instructions included in the PCR assay kit as described by Koskinen et al. [14]. The PCR assay can simultaneously identify the following 12 bacterial species or species groups: *Staphylococcus aureus*, *Staphylococcus* spp. (including *Staphylococcus aureus* and coagulase-negative staphylococci), staphylococcal β-lactamase gene, *Streptococcus agalactiae*, *Streptococcus dysgalactiae*, *Streptococcus uberis*, *Escherichia coli*, *Enterococcus* spp. (including *E*. *faecalis* and *E*. *faecium*), *Klebsiella* spp. (including *K*. *oxytoca* and *K*. *pneumonia*e), *Corynebacterium bovis*, *Serratia marcescens*, *Arcanobacterium pyogenes* and/or *Peptoniphilus indolicus*. The assay included 4 separate multiplex real-time PCR reactions, each detecting 3 bacterial species or species groups. Combination of the 4 different real-time PCR reactions results was the final result of one sample. The PCR test for the species-specific detection of the mentioned microorganisms has been shown to be suitable and to perform well, even when phylogenetically closely related non-targetspecies were tested [14]. The assay included enough controls for the validation of each run including negative DNA extraction controls, internal amplification standard (positive PCR controls), and non-template control that were included in each of the 4 real-time PCR reactions.

The PCR mixture consisted of 10 μl of the Master Mix, 5 μl of Primer Mix 1/2/3/4 (including species-specific primers and probes), and 10 μl of extracted DNA. The real-time PCR instrument thermal cycler Stratagene Mx3005P (Agilent Technologies Inc., Santa Clara, CA) was used for amplification. The amplification conditions were as follow: 95°C for 10 min,1 cycle; 95°C for 5 s and 60°C for 1 min, 40 cycles.

The real-time PCR fluorescence threshold was set, according to the assay’s Manual. When a Ct value was below 37, the reaction was positive. On the contrary, the reaction was negative. Positive and negative controls, and negative PCR control were included in each multiplex PCR reaction, for validation of the extraction and amplification process.

### Statistical analysis

The date of the occurrence of 11 mastitis pathogens and the staphylococcal β-lactamase gene in BTM samples and BTSCC were transferred to Microsoft Excel. We defined pathogen-positive by real-time PCR assay as "1" and pathogen-free by real-time PCR assay as "0". All statistical analyses were performed using SAS 9.3 (SAS Inst. Inc., Cary, NC). Data were analyzed as a factorial arrangement using PROC GLMMIX procedure of SAS with fixed effects of province and dairy cattle farming mode. The correlation between the BTSCC and the presence of mastitis pathogens were analyzed using PROC CORR procedure of SAS. All results were reported as least-square means (LSM). BTM sample was the experimental unit. The statistical model used as follow,

$${\text{Y}}_{\text{ijk}}=\mu +\gamma_{\text{i}}+\delta_{\text{j}}+{\left(\gamma \delta \right)}_{\text{ij}}+\varepsilon_{\text{ijk}}\;,$$

where Y_ijk_ was the proportion of the existence of mastitis pathogen, μ was overall mean, γ_i_ was province effect, δ←j was the dairy cattle farming mode effect, (γδ)_ij_ was the interaction between province effect and dairy cattle farming mode effect, and ε_ijk_ was random error. A *P*-value less than 0.0042 (0.05/12) was considered a significant correlation between the BTSCC and the presence of mastitis pathogens. Considering the repeated analysis, differences among effects were declared significant at *P* < 0.0042.

## Results

BTM samples from 894 dairy herds in 3 Chinese provinces were examined for mastitis pathogens. Proportion of BTM samples that were positive for 12 mastitis pathogen species or species groups are presented in Table 1. Among contagious pathogens, *S*. *aureus*, *S*. *agalactiae*, and *S*. *dysgalactiae* were detected in 448 (50.1%), 824 (92.2%), and 647 (72.3%) samples, respectively. Among environmental pathogens, *E*. *coli*, *S*. *uberis*, *Enterococcus* spp., *Klebsiella* spp., *S*. *marcescens*, *C*. *bovis*, and *A*. *pyogenes* were detected in 255 (28.6%), 79 (8.9%), 319 (35.7%), 178 (20.0%), 12 (1.3%), 152 (17.0%), and 601 (67.2%) samples, respectively. 850 (95.1%) samples were tested positive for *Staphylococcus* spp., 551 (61.7%) samples were tested positive for staphylococcal β-lactamase gene, and 343 (38.4%) BTM samples were simultaneously detected positive for *S*. *aureus* and staphylococcal β-lactamase gene. Overall, 743 BTM samples (83.1%) out of the total 894 BTM samples were positive for 4 or more species of mastitis bacterial pathogens.

**Table 1:** Proportion of 894 bulk milk samples that were positive for bovine mastitis pathogens in China from April 2012 to October 2013.

Bacterial species or species groups	Number of positive samples (herds)	Positive proportion of samples (%)
***S*. *aureus***	448	50.1
***S*. *agalactiae***	824	92.2
***S*. *dysgalactiae***	647	72.3
***E*. *coli***	255	28.6
***S*. *uberis***	79	8.9
***Enterococcus* spp.**	319	35.7
***Klebsiella* spp.**	178	20.0
***Serratia marcescens***	12	1.3
***C*. *bovis***	152	17.0
***A*. *pyogenes***	601	67.2
***Staphylococcus* spp.**	850	95.1
**staphylococcal β-lactamase gene**	551	61.7

The geometric mean BTSCC of the 583 herds was 650,000 cells/ml. *S*. *agalactiae* was detected in almost all of the 583 BTM samples (97.9%). A significant correlation (*P* < 0.0001) was observed between the presence of *S*. *aureus* and high BTSCC (Table 2). There was a significant difference (*P* = 0.0019) in BTSCC between *S*. *aureus*-positive herds and -negative herds. The geometric mean BTSCC of *S*. *aureus*-positive herds was 595,000 cells/ml, which was higher than that of -negative herds (441,000 cells/ml). It was worth noting that a significant relationship (*P* = 0.0039) was found between *A*. *pyogenes*-positive herds and high BTSCC. A significant correlation (*P* = 0.0007) was found between the presence of staphylococcal β-lactamase gene and high BTSCC. There was no correlation between the presence of other pathogens and BTSCC (*P* > 0.0042). No difference was observed in BTSCC between other pathogens positive and negative herds (*P* > 0.0042).

**Table 2:** Correlations between the presence of mastitis pathogens and the mean bulk tank milk somatic cell counts, and differences in bulk tank milk somatic cell counts between the mastitis pathogens positive and negative herds.

Bacterial species or species groups	Number of herds (583)		Mean BTSCC^a^ (×10^4^/ml)		Correlation Coefficients	*P1*^d^	*P2*^e^
	Positive^b^	Negative^c^	Positive^b^	Negative^c^			
***S*.* aureus***	310	273	59.5	44.1	0.33643	< .0001*	0.0019*
***E*. *coli***	111	472	54.2	49.5	0.03696	0.6384	0.366
***Staphylococcus* spp.**	529	54	49.4	54.3	0.10149	0.196	0.5561
***Klebsiella* spp.**	57	526	55.7	47.9	0.05035	0.522	0.2583
***Serratia marcescens***	9	574	40.6	63.1	-0.12319	0.1161	0.1659
***S*. *agalactiae***	574	9	59.1	44.5	0.15046	0.0545	0.4636
***Enterococcus* spp.**	142	441	49.6	54.1	-0.06643	0.398	0.3421
***S*. *uberis***	77	506	53.6	50.0	0.12429	0.1128	0.5521
***S*. *dysgalactiae***	543	40	46.2	57.5	0.05295	0.5007	0.223
***C*. *bovis***	128	455	51.6	52.1	0.06596	0.4014	0.9196
***A*. *pyogenes***	486	97	59.7	44.0	0.22438	0.0039*	0.0185
**β-lactamase**^f^	358	225	54.9	48.8	0.26074	0.0007*	0.2226

^a^BTSCC = bulk tank milk somatic cell counts.

^b^Positive: positive by real-time PCR assay.

^c^Negative: negative by real-time PCR assay.

^d^*P1*: correlations *P* value.

^e^*P2*: differences *P* value.

^f^β-lactamase = staphylococcal β-lactamase gene.

*P < 0.0042.

There were significant differences (*P* < 0.0001) in the presence of *S*. *aureus*, *S*. *agalactiae*, and *S*. *dysgalactiae* in BTM between samples collected from the small household farms, dairy-farming communities, and large-scaled dairy farms (Table 3). The prevalence levels of the three mastitis pathogens in dairy-farming communities were higher than those on small household farms and large-scaled dairy farms. The dairy herd prevalence of mastitis-causing bacteria showed significant geographic variation (Table 4). There were significant differences (*P* < 0.0042) in the presence of *S*. *agalactiae*, *S*. *dysgalactiae*, *A*. *pyogenes*, staphylococcal β-lactamase gene, Staphylococcus spp., *Klebsiella* spp., *Enterococcus* spp., and *S*. *uberis* in BTM samples among the 3 Chinese provinces.

**Table 3:** Effect of dairy cattle farming modes on the presence of mastitis pathogens.

Bacterial species or species groups	SHF^a^	DFC^b^	LSDF^c^	SEM^d^	*P*
***S*.* aureus***	0.5199^f^	0.6593	0.3804	0.0469	< .0001*
***E*. *coli***	0.3232	0.2363	0.2718	0.0433	0.4314
***Staphylococcus* spp.**	0.9802	0.9146	0.9157	0.0229	0.0890
***Klebsiella* spp.**	0.3066	0.1895	0.2240	0.0380	0.1227
***Serratia marcescens***	0.0170	0.0044	0.0152	0.0117	0.7394
***S*. *agalactiae***	0.6101	0.9683	0.8126	0.0244	< .0001*
***Enterococcus* spp.**	0.3733	0.2852	0.3284	0.0438	0.4330
***S*. *uberis***	0.1446	0.0886	0.1177	0.0295	0.4744
***S*. *dysgalactiae***	0.5797	0.8072	0.7589	0.0344	< .0001*
***C*. *bovis***	0.1634	0.1594	0.1397	0.0363	0.8652
***A*. *pyogenes***	0.7162	0.7585	0.6973	0.0413	0.5457
**β-lactamase**^e^	0.6776	0.6385	0.5259	0.0453	0.0298

^a^SHF = small household farm.

^b^DFC = dairy-farming community.

^c^LSDF = large-scaled dairy farm.

^d^SEM = standard error of the mean.

^e^β-lactamase = staphylococcal β-lactamase gene.

^f^0.5199 = the values in each column represent the proportion of the existence of mastitis pathogens

*P < 0.0042.

**Table 4:** Effect of regions on the presence of mastitis pathogens.

Bacterial species or species groups	Hebei	Heilongjiang	Inner Mongolia	SEM^a^	*P*
***S*.* aureus***	0.4431^c^	0.5492	0.5674	0.0366	0.1466
***E*. *coli***	0.2629	0.227	0.3414	0.0347	0.1415
***Staphylococcus* spp.**	0.9413	0.8746	0.9945	0.0441	0.0008*
***Klebsiella* spp.**	0.2129	0.1587	0.3486	0.0383	0.0009*
***Serratia marcescens***	0.0100	0.0238	0.0027	0.0416	0.4261
***S*. *agalactiae***	0.6243	0.7921	0.9746	0.0246	< .0001*
***Enterococcus* spp.**	0.3216	0.2302	0.4351	0.0473	0.0031*
***S*. *uberis***	0.1702	0.146	0.03466	0.0118	0.0018*
***S*. *dysgalactiae***	0.7408	0.8762	0.5289	0.0298	< .0001*
***C*. *bovis***	0.1404	0.1635	0.1586	0.0437	0.9035
***A*. *pyogenes***	0.8956	0.7079	0.5685	0.0457	< .0001*
**β-lactamase**^b^	0.6375	0.4079	0.7966	0.0231	< .0001*

^a^SEM = standard error of the mean.

^b^β-lactamase = staphylococcal β-lactamase gene.

^c^0.4431 = the values in each column represent the proportion of the existence of mastitis pathogens

*P < 0.0042.

## Discussion

In the current study, the multiplex real-time PCR assay in conjunction with FTA technique was used for the first time in China to estimate the herd prevalence of major mastitis pathogens in BTM from 894 China dairy herds. The relationships between the presence of mastitis pathogens and BTSCC, as well as the impacts of dairy cattle farming modes and region on bacterial species in BTM were investigated.

### FTA Card Technique and Real-Time PCR Assay

Because the areas of origin of the milk samples analyzed in this study were a great distance from the lab where they were analyzed, and due to the difficulty of guaranteeing the cold chain during transport, samples were inoculated and transported in FTA cards. BTM samples collected using FTA cards are easy to transport and face fewer shipping restrictions between the collection site and the laboratory without the need for a cold chain or liquid media handling [18]. The storage of BTM samples on FTA cards are also at room temperature and no refrigerators or freezers are required, which significantly reduces storage costs and space. The manufacturer states that genomic DNA entraped in FTA card matrix fibre which stored at room temperature is stable for 17 years [18]. The FTA cards are impregnated with special chemicals that inactivate infectious bacteria and viruses, reduce the potential biohazards of samples, and avoid cross-contamination between samples [16, 25]. As the FTA card contains proprietary chemicals that lyse cells and inactivate bacteria while preserving nucleic acids, BTM samples collected with FTA cards cannot be used for the culture and isolation of mastitis pathogens, and are suitable for molecular diagnostics. Furthermore, the FTA card is small (7.3 cm×3.8cm) and can be shipped in a standard letter mail envelope, which is difficult for liquid based medium. The FTA card technique simplifies the DNA extraction and purification procedures and is a promising tool for BTM samples collection, transportation, storage, and DNA preparation for mastitis pathogens.

The real-time PCR has proved to be a valuable diagnostic technique for improving bacterial identification and furthered benefits to shorten throughput time, to allow fully objective interpretation of data, and to quantify the number of microorganism in milk samples[13–14]. Previous studies revealed that the real-time PCR assay has higher sensitivity and specificity for diagnosis of mastitis pathogens compared with bacterial culture[15, 26–27]. As with all methodologies, the real-time PCR assay need thorough validation, particularly to avoid false positive or negative results due to the capability of detecting low copy numbers of bacteria originated from environment or inhibition of the reaction induced by components present in the sample. In the study of Koskinen et al., false positive results was detected in real-time PCR in the quarter milk samples originating from clinically healthy cows with a low milk SCC[28]. In addition, the real-time PCR assay is limited to detecting only the bacterial species for which a test kit has been designed and the clinical relevance of detecting bacterial nucleic acids instead of viable cells should be evaluated[28]. Despite all this, the real-time PCR assay is still a promising molecular method for detecting of mastitis pathogens.

This study used the PathoProof Mastitis Complete-12 KingFisher Kit (Finnzymes Oy, Espoo, Finland) to identify all major pathogens responsible for bovine mastitis from BTM samples. The PathoProof Mastitis PCR Assay, including all reagents for DNA extraction and the real-time PCR, is a commercially available PCR-based mastitis test. The assay can be used to extract DNA directly from raw or preserved milk, and it was developed to identify a total of 11 major mastitis-causing pathogens and the β-lactamase gene responsible for staphylococcal penicillin resistance [3, 14]. The assay was validated by Koskinen et al.[14], who demonstrated that the assay has 100% specificity and 100% sensitivity in identification of a large collection of culture strains originating from bovine mastitis. Taponen et al. [26] showed that the assay was able to identify mastitis bacteria in clinical mastitis samples with no bacterial growth in conventional culture. The assay has been used for identification of mastitis pathogens in all Danish dairy herds in November 2009 to January 2010 [3]. One disadvantage of the kit is that the real-time PCR assay is limited to detecting only the bacterial species without detecting *Mycoplasma bovis*. The PathoProof Mastitis Major-3 Kit used for identifying three contagious major mastitis pathogens including *M*. *bovis*, *S*. *aureus* and *S*. *agalactiae*, was recently reported [29]. In a word, the real-time PCR assay used in this study proved to be a useful tool for accurate diagnosis of mastitis-causing pathogens in BTM at routine milk recordings.

### Prevalence of Pathogens

This study indicated that contagious mammary pathogens are predominated among pathogens in BTM samples in China. Our findings for the dominance of contagious pathogens are not in accordance with following researches at herd level. Katholm et al. [3] tested BTM from all 4,258 Danish dairy herds using real-time PCR analysis. *Staphylococcus* spp. were found in all BTM, *S*. *uberis* in 95%, *C*. *bovis* in 90%, *Enterococcus* spp. in 78%, *A*. *pyogenes* and/or *P*. *indolicus* in 63%, and *E*. *coli* in 61%, whereas *S*. *agalactiae*, *S*. *aureus*, and *S*. *dysgalactiae* were found in 7, 91, and 86% of the herds. Rysanek et al. [1] cultured BTM Samples from 268 randomly selected herds and found dominance of environmental pathogens over the contagious pathogens in the Czech Republic. The isolation rates of *S*. *uberis*, *Enterococcus* spp., *E*. *coli*, *S*. *aureus*, *S*. *agalactiae*, and *S*. *dysgalactiae* in BTM samples were 18.3, 24.3, 19.0, 19.8, 5.6, and 17.9%, respectively. Phuektes et al. [6] analysed BTM from 42 herds in Australia with a multiplex PCR and reported that *S*. *uberis* was the most frequently present species in BTM, being found in 44% of samples, and *S*. *aureus*, *S*. *agalactiae*, and *S*. *dysgalactiae* were present in 16, 22 and 28% of BTM samples, respectively.

### Association between BTSCC and the presence of mastitis pathogens

The geometric mean of the BTSCC of China milk are higher compared with other industrialized countries [1, 6, 10]. In this study, there was a significant correlation between the presence of *S*. *aureus* and the high mean BTSCC in BTM. This is in agreement with previous studies [1, 5, 10, 30–33]. Riekerink et al. [10] reported a significant association between the mean BTSCC and the isolation of *S*. *aureus* from BTM samples. Wilson et al.[33] found that separate milking for cows infected with *S*. *aureus* reduced BTSCC from 600,000/ml to 345,000/ml. However, other studies showed that the mean BTSCC and detection of *S*. *aureus* were not significantly associated [31, 34]. Koop et al. [34] thought that the little effect on BTSCC was caused by the relatively low prevalence of infection with *S*. *aureus*, although udder infections with *S*. *aureus* can strongly increase quarter milk SCC.

Several studies showed that there was no significant relationship between the presence of environmental pathogens and BTSCC [1, 32]. This is in accordance with our results. In this study, no significant relationship were observed between BTSCC and the environmental mastitis-causing pathogen except *A*. *pyogenes*. It's worth noting that a significant relationship was found between the *A*. *pyogenes*-positive samples and the high BTSCC. Our results are in accordance with the findings of Klossowska et al. [35] and Malinowski et al. [36], who reported that the high SCC was significantly associated with intramammary infections by *A*. *pyogenes*.

### Differences in the prevalence of mastitis pathogens between dairy cattle farming modes

The dairy herd’s prevalence of mastitis-causing bacteria differed between the small household farms, dairy-farming communities and large-scaled dairy farms in our study. Significant differences (*P* < 0.0001) were found in the prevalence of *S*. *aureus*, *S*. *agalactiae*, and *S*. *dysgalactiae* in BTM between the different dairy cattle farming modes. The prevalence levels of the 3 mastitis pathogens in dairy-farming communities were higher than those on small household farms and large-scaled dairy farms. *S*. *agalactiae* and *S*. *dysgalactiae* occured less frequently in small household farms than that in dairy-farming communities and large-scaled dairy farms, whereas *S*. *aureus* occured less frequently in large-scaled dairy farms. Herd size and farm management practices had considerable influence on the prevalence of mastitis pathogens in BTM [5, 7, 37]. In dairy-farming communities, the dairy cattle were milked by owners of cows, therefore the healthy cows and infected cows were milked at the same time. This may transmit the contagious pathogens from infected cows to healthy cows via the milking machinery. Another possible reason for dairy-farming communities having higher prevalence levels of contagious pathogens may be due to higher levels of cattle movement. Herds that introduce new cattle could significantly increase the risk of being infected with contagious pathogens [37–38].

### Differences in the Prevalence of Mastitis Pathogens between Provinces

The dairy herd’s prevalence of mastitis-causing bacteria differed between provinces in our study. There were highly significant differences (*P* < 0.0042) in the prevalence of *S*. *agalactiae*, *S*. *dysgalactiae*, *A*. *pyogenes*, staphylococcal β-lactamase gene, *Staphylococcus* spp., *Klebsiella* spp., *Enterococcus* spp. and *S*. *uberis* in BTM between the 3 provinces. The differences are mainly caused by geographic variation. Riekerink et al. [39] found that the distribution of mastitis-causing bacteria showed substantial geographic variation on Canadian dairy farms. Although highly significant differences in the prevalence of mastitis-causing bacteria were observed between the 3 Chinese provinces, the most frequently detected mastitis-causing bacteria species were almost the same. In Inner Mongolia, *Staphylococcus* spp., *S*. *agalactiae*, *A*. *pyogenes*, and *S*. *aureus* were the most frequently detected bacteria from dairy herds, whereas in Hebei and Heilongjiang, the most often discovered pathogens were *Staphylococcus* spp., *S*. *dysgalactiae*, *S*. *agalactiae*, and *A*. *pyogenes*.

In this study, sampling BTM was performed only once. This may underestimated the prevalence of mastitis pathogens due to intermittent shedding. Multiple sampling of BTM from the same herd over a period of time is needed to determine the true prevalence of contagious mastitis pathogens [10, 40–41].

## Conclusions

The current study revealed that contagious mammary pathogens predominated in BTM samples in China. *Staphylococcus* spp., *S*. *agalactiae*, *S*. *dysgalactiae*, *A*. *pyogenes*, staphylococcal β-lactamase gene, and *S*. *aureus* were the most frequently detected pathogens in BTM samples. The presence of *S*. *aureus* and *A*. *pyogenes* were significantly associated (*P* < 0.0042) with the high BTSCC. There was a significant difference (*P* < 0.0042) in BTSCC between *S*. *aureus*-positive herds and *S*. *aureus*-negative herds. Significant differences (*P* < 0.0001) were found in the presence of *S*. *aureus*, *S*. *agalactiae*, and *S*. *dysgalactiae* in BTM between the small household farms, dairy-farming communities, and large-scaled dairy farms. The prevalence levels of the 3 mastitis pathogens in dairy-farming communities were higher than those on small household farms and large-scaled dairy farms. There were significant differences (*P* < 0.0042) in the presence of *S*. *agalactiae*, *S*. *dysgalactiae*, *A*. *pyogenes*, staphylococcal β-lactamase gene, *Staphylococcus* spp., *Klebsiella* spp., *Enterococcus* spp., and *S*. *uberis* in BTM between Inner Mongolia, Heilongjiang, and Hebei Province.
